# Data for molecular dynamics simulations of B-type cytochrome c oxidase with the Amber force field

**DOI:** 10.1016/j.dib.2016.07.043

**Published:** 2016-07-26

**Authors:** Longhua Yang, Åge A. Skjevik, Wen-Ge Han Du, Louis Noodleman, Ross C. Walker, Andreas W. Götz

**Affiliations:** a Department of Chemistry, Nanchang University, 999 Xuefudadao, Nanchang, Jiangxi 330031, China; b San Diego Supercomputer Center, University of California San Diego, 9500 Gilman Drive, La Jolla, CA 92093, USA; c Department of Biomedicine, University of Bergen, N-5009 Bergen, Norway; d Department of Integrative Structural and Computational Biology, GAC1118, The Scripps Research Institute, 10550 North Torrey Pines Road, La Jolla, CA, USA; e Department of Chemistry and Biochemistry, University of California San Diego, 9500 Gilman Drive, La Jolla, CA 92093, USA

## Abstract

Cytochrome *c* oxidase (CcO) is a vital enzyme that catalyzes the reduction of molecular oxygen to water and pumps protons across mitochondrial and bacterial membranes. This article presents parameters for the cofactors of ba_3_-type CcO that are compatible with the all-atom Amber ff12SB and ff14SB force fields. Specifically, parameters were developed for the Cu_A_ pair, heme b, and the dinuclear center that consists of heme a_3_ and Cu_B_ bridged by a hydroperoxo group. The data includes geometries in XYZ coordinate format for cluster models that were employed to compute proton transfer energies and derive bond parameters and point charges for the force field using density functional theory. Also included are the final parameter files that can be employed with the Amber leap program to generate input files for molecular dynamics simulations with the Amber software package. Based on the high resolution (1.8 Å) X-ray crystal structure of the ba_3_-type CcO from *Thermus thermophilus* (Protein Data Bank ID number PDB: 3S8F), we built a model that is embedded in a POPC lipid bilayer membrane and solvated with TIP3P water molecules and counterions. We provide PDB data files of the initial model and the equilibrated model that can be used for further studies.

**Specifications Table**

**Table:** 

Subject area	*Chemistry, Biology*
More specific subject area	*Biophysical, Computational chemistry*
Type of data	*PDB files, XYZ files and Amber parameter files*
How data was acquired	*Density functional theory calculations with ADF; model preparation with the CHARMM-GUI membrane builder, VMD and AmberTools; molecular dynamics simulations with Amber*
Data format	*XYZ coordinate files, PDB coordinate files, Amber OFF library files and parameter files*
Experimental factors	*Starting geometries based on crystal structure from Protein Data Bank ID number PDB:*3S8F
Experimental features	*Geometry optimization and ESP charge derivation of cluster models with PW91-D3 and OLYP exchange-correlation functionals; 10ns molecular dynamics equilibration with Amber ff12SB, GAFF, Lipid14 force fields in combination with new parameters and TIP3P water.*
Data source location	*SDSC, San Diego, CA, USA*
Data accessibility	*Data are within this article and via the Protein Data Bank ID number PDB:*3S8F.

## Value of the data

• The XYZ coordinates of the cluster models of the cytochrome c oxidase (CcO) cofactors can serve as starting points for further electronic structure calculations.
• Additional electronic structure calculations can be employed to derive charges and bond parameters for different redox states of the CcO cofactors.
• The Amber force field parameters are suitable to set up MD simulations of CcO in the parameterized redox states of the cofactors.
• The PDB coordinates of CcO embedded in lipid bilayer membrane and water are useful as starting point for additional simulations with different protonation states or mutations.

## 1 Data

The data provided in this article were generated for molecular dynamics (MD) simulations of ba_3_-type cytochrome c oxidase (CcO) of *Thermus thermophilus* [1]. Atomic coordinate data of cluster models of CcO cofactors are provided in XYZ coordinate format and atomic coordinate data of the entire CcO model embedded in POPC lipid bilayer and solvated with TIP3P water molecules and counterions are provided in PDB file format. Force field parameter data compatible with the Amber ff12SB or ff14SB force fields are provided in standard Amber OFF library and parameter format for the Cu_A_ pair including coordinating side chains; heme b; and the dinuclear reaction center (DNC), which consists of heme a_3_, Cu_B_ and coordinating residues. Scripts for automated building of the enzyme-membrane system with the leap program of the Amber software package are also provided.

## 2 Experimental design, materials and methods

### 2.1 Model preparation (PDB data file generation)

The CcO model that was employed for molecular dynamics simulations in Ref. [1] is based on the high resolution (1.8 Å) X-ray crystal structure of the ba_3_-type CcO from *Thermus thermophilus* in the reduced state in lipidic cubic phase (LCP) crystal as obtained from the Protein Data Bank (PDB: 3S8F) [2]. This crystal structure contains two oxygen atoms between the Fe and Cu centers of the DNC, which have been proposed to be a bridging hydroperoxide [3]. Our model keeps this bridging hydroperoxide and uses oxidation states of the cofactors (see Section 3) in which the heme b and Cu_A_ pair are reduced, that is Fe_b_^2+^ and Cu_A_^+^–Cu_A_^+^, while the DNC (heme a_3_ and Cu_B_) is fully oxidized with Fe_a3_^3+^ and Cu_B_^2+^. This model corresponds to state **6** in the CcO reaction cycle presented by Noodleman and coworkers [4], [5].

The H++ software [6] was used to determine the protonation states of titratable residues. H++ cannot recognize non-standard complex ligands such as heme automatically. We therefore manually supplied PQR files (PDB+charge+radius) with appropriate charges. For standard residues, the charges from the Amber ff12SB force field were used. The charges of cofactors were obtained through QM calculations as described in Section 3 of this article. General atomic van der Waals radii were used (C: 1.70 Å; N: 1.55 Å; Fe: 1.00 Å; O: 1.50 Å; Cu: 1.40 Å; H: 1.20 Å). The pH was set to 6.5. All Arg, Tyr and Lys residues are predicted to be protonated with high pKa values (Arg>11.7, Tyr>8.9, Lys>8.7). All but two Glu residues are predicted to have pKa values <5.4, while Glu203 and Glu131^II^ have pKa values of 7.0 and 6.7, respectively. We have assumed that all Glu residues are in their standard, deprotonated state. All Asp residues apart from Asp372 have pKa values <4.1 and are thus deprotonated, while Asp372 is protonated. Most His residues have rather low pKa values while His462, His552, and His8^II^ have pKa values around 6.7 and His376 has a pKa>12. The PDB files provided here have all His residues in their default protonation state (singly protonated on the ε-nitrogen atom), including His376. This corresponds to protonation state A in Ref. [1]. The lowest energy protonation state would have a doubly protonated His376 (state D in Ref. [1]).

The protein including 189 X-ray crystal water molecules was inserted into a palmitoyl-oleoyl-phosphatidylcholine (POPC) bilayer of 180 lipid molecules and solvated in 0.15 M KCl solution with 18,866 water molecules, followed by removal of K^**+**^ ions to neutralize the system. The CHARMM-GUI membrane builder [7], VMD [8], and the AmberTools [9], [10] charmmlipid2amber.py program were used as described in Ref. [1]. The resulting PDB data that is provided with this article contains coordinates for protonation state A of Ref. [1]. We provide both the initial coordinates for our model that are based on the crystal structure (cco-lipid-ion-water-crystal.pdb) and coordinates obtained through equilibration using an MD protocol (see Ref. [1]) with the newly developed parameters (cco-lipid-ion-water-equilibrated.pdb). Other protonation states or mutants can be easily generated by appropriate renaming of residues in the PDB files. The example build scripts that are provided with this article can be employed by the AmberTools leap program to set up input files for MD simulations with Amber. The build scripts generate files with the Amber Lipid14 force field [11] in combination with the Amber ff12SB force field [12], TIP3P parameters for water [13], the Joung/Cheatham ion parameters [14], and the parameters for non-standard residues that are provided with this article.

## 3 Parameter derivation for CcO cofactors (cluster model XYZ data, Amber parameter data)

Force field parameters for the Cu_A_ pair (cua-pair.prm), heme b (heme.prm), and the DNC heme a_3_ and Cu_B_ (heme.prm, dnc.prm) were obtained as described in Ref. [1] and detailed below. Parameters for heme b including bonded parameters to the coordinating histidine residues and Lennard–Jones parameters for Cu are based on literature data [15], [16]. Parameters for heme a_3_ are derived from these heme b parameters and combined with GAFF [17] parameters for the geranyl–geranyl tail and the formyl group. GAFF parameters are also used for the bridging hydroperoxide in the DNC and the special C–N bond between His233 and Tyr237. Harmonic bond and angle constraints are used between all metal centers and the ligands, with values that are based on the crystal structure for the Cu_A_ pair and based on geometries optimized for cluster models with density functional theory (DFT) for the DNC.

### 3.1 DNC cluster model geometry optimization

DFT geometry optimizations were performed with the ADF program [18], [19], [20] using the OLYP exchange-correlation functional [21] and double- and triple-zeta polarized (DZP and TZP) Slater type basis sets (TZP on the metal atoms and DZP on all other atoms) from the ADF basis set library [22]. The COSMO model [23], [24], [25] with a dielectric constant of *ε*=18.5 was used to approximate the effect of the protein environment. The geometry optimizations of the DNC employed the same 205 atom cluster model (opt-DNC-full-model.xyz) used previously by Noodleman et al. [4] It contains heme a_3_, the side chain of the axial His384 ligand to heme Fe_a3_, the bridging hydroperoxide, Cu_B_ and the side chains of its three coordinating ligands His283, His282 and the special His233 that is covalently linked to Tyr237, as well as the side chains of the three residues Arg449, doubly protonated His376 (positively charged, +1) and protonated Asp372 above the DNC, Gly232 including backbone atoms linked to His233, and 7 water molecules. The low spin Fe^3+^ and Cu^2+^ centers of the DNC were antiferromagnetically coupled and the positions of the link atoms were constrained to the crystal structure coordinates during the DFT geometry optimizations.

### 3.2 Cofactors charge derivation

Charges for the cofactors were computed with DFT using the OLYP potential and an electrostatic potential (ESP) fit approach as implemented in the SCRF module [26], [27] of ADF. For the Cu_A_ pair we employed a 36 atoms cluster model (charge-derivation-cua-pair.xyz). It includes the two Cu atoms and the side chains of the coordinating histidine and cysteine residues His114^II^, His157^II^, Cys149^II^, and Cys153^II^. For heme b we used a 97 atoms cluster model (charge-derivation-hemeb.xyz) that includes both the heme b and the side chains of its two axial histidine ligands, residues His72 and His386. For the DNC, we employed the coordinates from the geometry optimized cluster model, added the geranyl–geranyl tail and removed residues Arg449, His376, Asp372 and Gly232. We generated two different charge sets for the DNC, one with deprotonated PRAa_3_ (192 atoms, charge-derivation-DNC.xyz), and one with protonated PRAa_3_ (193 atoms, charge-derivation-DNC-PRAa_3_H.xyz).

The ESP charges are used both for the cofactors and the coordinating residues, which thus carry charges that differ from the standard AMBER ff12SB charges. Charges on symmetry equivalent atoms were averaged and all charges were scaled uniformly to yield the correct integer charge. Data with residue definitions and these optimized charges are contained in the Amber OFF format library files for the Cu_A_ pair (cua-pair.lib), heme b (hemeb.lib), and the DNC (dnc.lib).

## 4 Proton transfer energies (cluster model XYZ data)

Cluster models of the DNC (204 atoms, 205 if protonated) were developed for the purpose of computing reaction energies for the protonation of PRAa_3_ and proton transfer to His376 [1] (see also Ref. [28]) and were optimized with DFT as described above, however, employing the PW91-D3 exchange-correlation functional including dispersion correction. [29], [30] The inner cores of C(1s), N(1s), O(1s), Fe(1s,2s,2p), and Cu(1s,2s,2p) are treated by the frozen core approximation. The optimized coordinate data is supplied in XYZ file format (opt-DNC-state-Aprime.xyz, opt-DNC-state-Cprime-1.xyz, opt-DNC-state-Cprime-2.xyz, opt-DNC-state-D.xyz).
